# Report of the Chicago Charitable Eye and Ear Infirmary

**Published:** 1860-06

**Authors:** 


### SECOND ANNUAL REPORT
### OF THE
### CHICAGO CHARITABLE EYE AND EAR INFIRMARY, FOR THE YEAR ENDING MAY 1, 1860.

OFFICERS OF THE ASSOCIATION.—PRESIDENT, Walter L. Newberry.

VICE PPESIDENTS—Charles V. Dyer, Luther Haven.

SECRETARY—Samuel Stone.

TREASURER—Edward L. Holmes.

TRUSTEES—Walter L. Newberry, William H. Brown, Chas. V. Dyer, Luther Haven, Ezra B. McCagg, William Barry, Flavel Moseley, Samuel Stone, Philo Carpenter, Rev. N. L. Rice, D. D., John H. Kinzie, Mark Skinner.

BOARD OF SURGEONS, (Trustees ex-officio.)—CONSULTING SURGEONS.—Prof. Daniel Brainard M. D., Prof. Joseph W. Freer, M. D.

ATTENDING SURGEONS.—Edward L. Holmes, M. D., Edwin Powell, M. D.

It must be a matter of interest to our readers to learn that the Infirmary continues in a flourishing condition. During the past year *one hundred and seventy-seven* patients have been under treatment, making an aggregate of *two hundred and ninety-two* patients, since the establishment of the Infirmary, two years since. These patients have all been from the poor and destitute classes of society.

Although but a small portion of the public are yet aware of the existence of the Infirmary, the number of its patients has been constantly increasing.

In a city as large as Chicago, an Infirmary for the poor, afflicted with diseases of the eye, should be maintained at public expense, since it is more in accordance with an enlightened humanity, and it is more politic and economical to furnish the destitute with gratuitous treatment than to support them in our Blind Asylums, after they have lost the power of vision.

For the purpose of preventing, as far as possible, blindness and its attending evils, several States have granted considerable sums of money for the establishment and support of charitable Eye Infirmaries. The annual reports of these institutions, show what incalculable good they may accomplish. Many patients, in our Eastern cities, are wholly indebted to Eye Infirmaries for the preservation of their sight, thus being saved the painful necessity of becoming a burden either to their friends or to the public at large.

There is another reason, and a very important one, why an Institution of this kind should recommend itself to the public, and receive the encouragement of our profession. An Eye

Infirmary, under the charge of competent surgeons, will afford ample opportunities for clinical instruction in diseases of the eye, which are among the most important affections we are called upon to treat. It is universally admitted that this department of medical science has been too much neglected by the physicians of this country. We believe the establishment of good Infirmaries in all our cities, where medical instruction is sought by the student, would tend to awaken a greater interest in this important subject. An Infirmary, in which the student can obtain a thorough knowledge of the theory and practice of ophthalmic medicine, will, indirectly, accomplish more good, perhaps, than it can by the direct benefits conferred upon the patients under treatment. Whatever contributes to the advancement of a knowledge of ophthalmic science among our practitioners, must be the source of inestimable good to the public.

We trust the Infirmary will receive the encouragement and support of the public, and especially of the profession.

The Second Annual Report of the Surgeons of the Infirmary is worthy of a careful perusal, as showing the practical utility of such an institution.

